# Coefficient of Energy Balance: Effective Tool for Early Differential Diagnosis of CNS Diseases

**DOI:** 10.1155/2013/745943

**Published:** 2013-06-20

**Authors:** Klára Bořecká, Pavel Adam, Ondřej Sobek, Lenka Hajduková, Věra Lánská, Petr Nekola

**Affiliations:** ^1^Department of Clinical Biochemistry, Thomayer's Hospital, Vídeňská 800, 140 59 Prague, Czech Republic; ^2^Laboratory for CSF and Neuroimmunology, Topelex Ltd, Central Military Hospital Complex, U Vojenské nemocnice 1200, 162 00 Prague, Czech Republic; ^3^Department of Neurology, 3rd Medical Faculty, Charles University, Šrobárova 50, 100 34 Prague, Czech Republic; ^4^Statistical Unit, Institute for Clinical and Experimental Medicine, Vídeňská 1958/9, 140 21 Prague, Czech Republic

## Abstract

Urgent examination of cerebrospinal fluid (CSF) provides immediate important information about the character of central nervous system (CNS) impairment. Although this examination includes energy parameters such as glucose and lactate concentrations, it does not commonly use Coefficient of Energy Balance (CEB). In this study, we focused on CEB because it enables more exact assessment of actual energy state in the CSF compartment than glucose and lactate alone. CEB informs about the actual functioning condition of present cells, and it does not require any other analysis or costs. Using Kruskal-Wallis ANOVA, we examined a large CSF sample (*n* = 8183) and we compared CEB values among groups with different cytological syndromes. We found a statistically significant difference of CEB between the group with granulocyte pleocytosis and the control group. These results indicate a high degree of anaerobic metabolism caused by the oxidative burst of neutrophils. Similarly, we found a statistically significant difference of CEB between the control group and groups with tumorous oligocytosis plus pleocytosis and monocyte pleocytosis. This difference can be attributed to the oxidative burst of macrophages. Our findings suggest that CEB combined with CSF cytology has a great importance for diagnosis, differential diagnosis, and early therapy of CNS diseases.

## 1. Introduction

Unlike blood analysis, an examination of CSF allows the following of immune and metabolic processes in CNS without disturbing other organ systems as the CSF compartment is considerably autonomous. The urgent (fundamental) examination of CSF should be accessible everywhere 24 hours a day to provide important information in the decision-making diagnostic and therapeutic algorithms [1, 2]. The urgent CSF examination includes an analysis of energy balance, determination of blood-CSF barrier permeability, spectrophotometric analysis, total cell number and cytological investigation, and possibly a measurement of “quick” destruction markers. The examination should be complemented by an analysis of CRP (C-reactive protein) concentration in blood to give a picture of the system inflammation state [1–7]. 

Biochemical parameters of energy metabolism in the CSF compartment typically comprise a concentration of the energetic substrate, that is, glucose in CSF and blood, glucose quotient *Q*_glu_(*Q*_glu_ = ⌊glucose_CSF_⌋/[glucose_blood_]), and concentration of the product of anaerobic metabolism, that is, lactate in CSF [2, 4]. Glucose is actively transported across blood-CSF barrier; therefore, its CSF concentration is dependent on concentration in serum [8]. An importance of lactate for distinction of bacterial meningitis from aseptic/viral meningitis is well known [9–11], as well as a prognostic usefulness of lactate in many CNS diseases [12]. Because the concentration of lactate is substantially influenced by an amount of glucose that comes in the metabolic processes, it is not the absolute indicator of an anaerobic metabolism extent. The Coefficient of Energy Balance (CEB) links consumption of glucose, production of lactate, and production of ATP to one mutual mathematical formula [6]; therefore, the inverse relationship of lactate and glucose concentrations multiplies the sensitivity of CEB. CEB accurately and sensitively evaluates the actual anaerobic metabolism extent in a territory adjacent to the CSF compartment [5–7, 13–16]. Unlike glucose or lactate, CEB can stratify a metabolic turnover to more layers corresponding with different diseases. CEB has been described in local studies [5–7, 13–16], but it has not been widely used and well known yet. According to recent studies the Coefficient of Energy Balance correlates better (*ρ* = −0.770) with CSF cellularity than lactate or glucose concentrations in purulent meningitis [15], and CEB value below 10.00 has 100.0% sensitivity and 92.1% specificity for diagnosis of purulent meningitis [13]. An evaluation of metabolic turnover in the CSF compartment by CEB in combination with number of cells and cytology enables the determination of the features and intensity of inflammation. Thus CEB has a crucial importance for differentiating the cause of CNS disability [15].

To calculate CEB, it is important to understand the processes of energetic metabolism of glucose. Glucose as an energy substrate is changed by glycolysis to pyruvate in CNS. If a sufficient amount of oxygen is dissolved in CSF compartment, the glycolysis takes place primarily in an *aerobic* way. In aerobic glycolysis pyruvate is further metabolized by the Krebs cycle and the respiratory chain to CO_2_ and H_2_O producing 38 molecules of adenosine triphosphate (ATP) from 1 molecule of glucose. If the amount of oxygen in the CSF compartment is low, the extent of *anaerobic* glycolysis increases. In anaerobic glycolysis pyruvate is converted to lactate producing only 2 ATP molecules from 1 molecule of glucose [4, 13]. Obviously, this energy state is disadvantageous, especially for CNS tissue. The Coefficient of Energy Balance [6] is a mathematical expression of the described biochemical processes:

(1) $$\begin{array}{c} \text{CEB}=38-18*\frac{\left\lfloor {\text{lactate}}_{\text{CSF}}\right\rfloor }{\left[{\text{glucose}}_{\text{CSF}}\right]}. \end{array}$$

CEB determines the average number of ATP molecules produced from 1 molecule of glucose in current circumstances in the CSF compartment and thus expresses the proportion of anaerobic glucose metabolism [5–7, 13–16].

Under the physiological conditions in CSF, the considerable amount of oxygen is dissolved there and a natural consumption of glucose is continuously supplied from blood. As a result, concentration of glucose is quite high (*Q*_glu_ more than 0.55), concentration of lactate is low, and value of CEB within aerobic metabolism is in the reference range, that is, 28.00–38.00 [3, 5, 13, 14]. 

Serous inflammations can be either of infectious (e.g., viruses, *Treponema pallidum, *and *Borrelia* sp.) or noninfectious etiology (autoimmune diseases, paraneoplastic impairment, after cerebral bleeding, during reparations, and regeneration of damaged CNS tissue). When serous CNS inflammation occurs, a permeability of blood-CSF barrier increases, humoral immune components and cells penetrate CSF, and activated immune system raises energy demands. Limited tissue reserves of glucose [11] may cause an advanced extent of anaerobic metabolism in cerebrospinal fluid. In consequence, concentration of glucose slightly decreases (*Q*_glu_ under 0.55), concentration of lactate slightly increases, and value of CEB ranges from 10.00 to 28.00 [5–7, 13–16]. 

**Figure 1:**
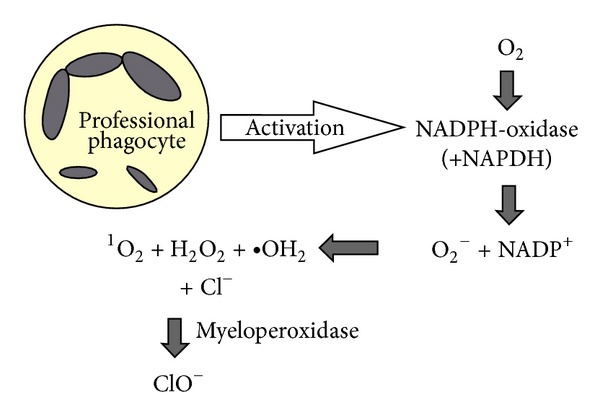
The oxidative burst of professional phagocytes (neutrophils, macrophages): the oxidative burst is induced by activation of NADPH-oxidase producing reactive oxygen species, by which phagocytes destroy devoured pathogens. Then myeloperoxidase catalyzes oxidation of chlorides to high bactericidal hypochlorites with participation of hydrogen peroxide [5, 22–28].

Diametrical difference in energy state is caused by the so-called “oxidative (respiratory) burst of professional phagocytes” when purulent CNS inflammation or another kind of pathologies occurs. See Figure 1.

During this process a consumption of oxygen is substantial, so an intensive anaerobic metabolism of glucose is developed, and as a result, concentration of glucose in CSF significantly decreases (or *Q*_glu_), concentration of lactate significantly increases, and value of CEB distinctly falls, ranging between 10.00 and highly negative values [5–7, 13–16]. Professional phagocytes may be neutrophils in case of purulent inflammation (of infectious etiology—usually extracellular bacteria—or noninfectious etiology—after a reperfusion of ischemic parts incurred as a consequence of vasospasms after subarachnoid hemorrhage) [5–7, 13–16, 29–33]. Professional phagocytes may be also macrophages, in case of an elimination of intracellular bacteria and yeasts or molds (*Mycobacterium tuberculosis, Listeria monocytogenes, *rarely *Borrelia *sp.*, Treponema pallidum, Cryptococcus neoformans, *and *Candida albicans*) or at the malignant meningeal infiltration, MMI [5–7, 13–16]. This means that CEB values from 10.00 to highly negative values are usually accompanied by granulocyte pleocytosis (the oxidative burst of neutrophils) and minor tumorous pleocytosis or oligocytosis, monocyte pleocytosis or oligocytosis, or rarely lymphocyte pleocytosis or oligocytosis (the oxidative burst of macrophages). An adherence of macrophages to tissue—an epicenter of pathological process—may sometimes cause a minority representation of monocyte-macrophage cells in CSF, while lymphocytic cells predominate in cerebrospinal fluid. A high scope of anaerobic metabolism in the cerebrospinal fluid compartment should evoke a suspicion of an infection caused by the above-mentioned microbes or CNS cancer.

In this study we focused on CEB because it has a greater information potential than glucose or lactate itself. CEB enables one to accurately express the range of anaerobic glucose metabolism, and it can stratify a metabolic turnover. It is commonly and early available within a fundamental (urgent) CSF examination, without the necessity of any other analysis or costs. We examined a large CSF sample from patients with variant neurological symptoms and compared CEB values among eight cytological groups to match the findings with the above-mentioned studies. We hypothesized that values of CEB would be significantly different in groups accompanied by the oxidative burst of professional phagocytes. This means that significantly different CEB would be in the group with granulocyte pleocytosis or tumorous pleocytosis/oligocytosis, possibly monocyte pleocytosis. Our results confirm this hypothesis. They suggest that CEB, commonly assessed with CSF cytology, can differentiate between variant pathologies in CNS specify and accelerate differential diagnosis and early targeted therapy of CNS diseases.

## 2. Materials and Methods

We analyzed 8183 CSF samples in the Laboratory for CSF and Neuroimmunology (the Expert laboratory in CSF analysis in Czech Republic, http://www.likvor.cz). In all cases, physicians requested this examination because of a suspicion of some neurological disease (there were signs of meningeal irritation, focal neurological findings, febrile disorders, disturbances of consciousness, complications after head injury or neurosurgical operations, etc.). Firstly we examined a total cell number in the Fuchs-Rosenthal chambre, basic biochemistry, secondly we made a qualitative cytological examination, and thirdly we assumed levels of IgG, IgA, IgM, and their CSF/S quotients, and β2-m in CSF and serum and its CSF/S quotient. In the cytological examination we used standard (MGG = May-Grünwald + Giemsa-Romanovski) and special staining techniques in an effort to detect malignant elements (toluidine blue stain, Papanicolaou) and the presence of CNS tissue destruction (Oil Red 0) by detecting of lipophagic macrophages. Cytological samples were obtained from CSF using a gentle sedimentation technique. With immunoglobulins, their intrathecal oligoclonal synthesis, if existent, was determined numerically (Reiber).

According to the findings in cytology we divided the sample into 8 groups: control group (*n* = 235), granulocyte oligocytosis (GO, *n* = 64), granulocyte pleocytosis (GP, *n* = 766), monocyte oligocytosis (MO, *n* = 2699), monocyte pleocytosis (MP, *n* = 1457), lymphocyte oligocytosis (LO, *n* = 1200), lymphocyte pleocytosis (LP, *n* = 1610), and tumorous oligocytosis plus pleocytosis (TO + TP, *n* = 152). We included TO in the same group as TP because of small sample size and the same etiology. *Pleocytosis *is determined as the number of leukocytes in the Fuchs-Rosenthal chamber greater than 4/1 *μ*L; *oligocytosis* as the number of leukocytes in the Fuchs-Rosenthal chamber in reference range (to 4/1 *μ*L), but with a pathological cell composition—the attributive adjective according to the predominant cells (except TP/TO, where the adjective tumorous is in case of any presence of malignant cells in CSF).

We defined the control group as a group with results of biochemical and cytological CSF analysis in the reference range, including beta-2-microglobulin concentration in CSF and zero intrathecal synthesis in classes IgG, IgA, and IgM according to Reiber's calculation—see decision limits in Table 1. Although these criteria may not be considered to be fully convincing, there is virtually no other way of further differentiation (we do not perform lumbar puncture in healthy people). Samples with parameters not meeting the above criteria were automatically regarded as samples from patients with pathological CSF findings.

Concentrations of glucose in CSF and serum, total protein in CSF, and lactate in CSF were analyzed photometrically (Synchron Lx by Beckman; Viva by Siemens); concentration of beta-2-microglobulin_CSF_ was analyzed by nephelometry (Array by Beckman, BN II by Siemens); *Q*_glu_ and CEB were calculated.

Descriptive methods, Kolmogorov-Smirnov test, box-and-whiskers plots, and Kruskal-Wallis ANOVA with pairwise posthoc multiple comparisons were used for statistical analysis.

## 3. Results and Discussion

Descriptive statistics of CEB with individual cytological syndromes in cerebrospinal fluid and results of testing of concordance among 8 groups are summarized in Tables 2 and 3.

**Figure 2:**
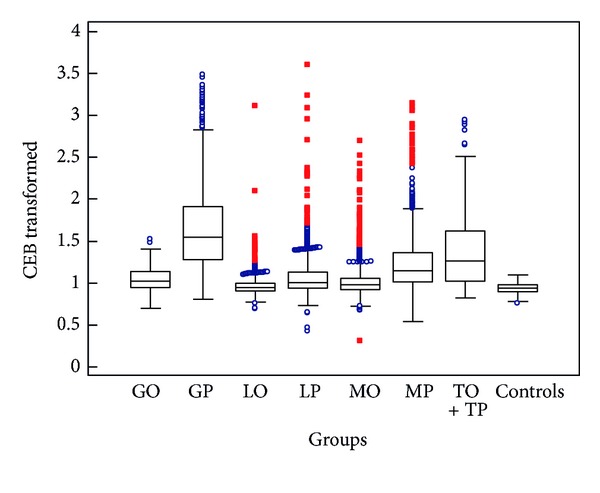
CEB with defined cytological syndromes in CSF (box-and-whiskers plots; GO: granulocyte oligocytosis, GP: granulocyte pleocytosis, LO: lymphocyte oligocytosis, LP: lymphocyte pleocytosis, MO: monocyte oligocytosis, MP: monocyte pleocytosis, TO + TP: tumorous oligocytosis + pleocytosis).

**Figure 3:**
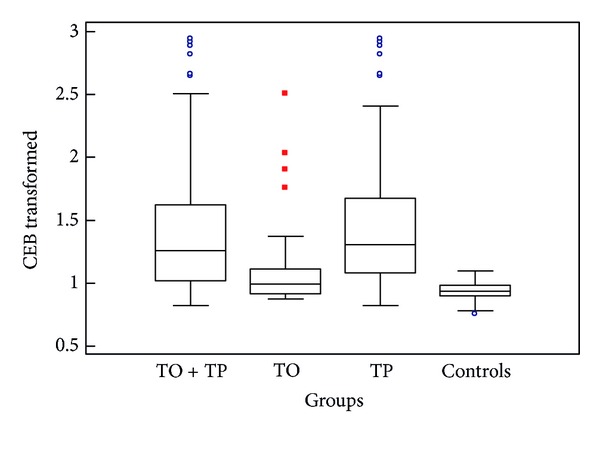
CEB with tumorous pleocytosis and tumorous oligocytosis (box-and-whiskers plots, CEB in the common TO + TP group versus separated TO and TP groups; TO: tumorous oligocytosis, TP: tumorous pleocytosis).

Because the distribution of CEB in our data set is not normal and CEB values are often strongly negative, we performed the transformation to CEB_transf_(CEB_transf_ = log⁡(40 − CEB)) for creation of box-and-whiskers plots—see Figures 2 and 3. 

As can be seen from Tables 2 and 3 and Figure 2, we found a statistically significant difference of CEB between the group with granulocyte pleocytosis and the control group (*P* < 0.001), respectively, significantly decreased values of CEB, as well as in comparison with all other cytological groups, that is, LO, LP, MO, MP, TO + TP and GO (*P* < 0.001 or *P* < 0.01). Similarly, the group with TO + TP presents the statistically significant difference of CEB from controls (*P* < 0.001) and other cytological groups (*P* < 0.001 or *P* < 0.01), except for MP (between TO + TP and MP is no statistical difference). Consequently, our results support the above-mentioned theory of the oxidative burst of professional phagocytes and probability of simultaneous occurrence of anaerobic scope of CEB and certain cytological patterns.

**Figure 4:**
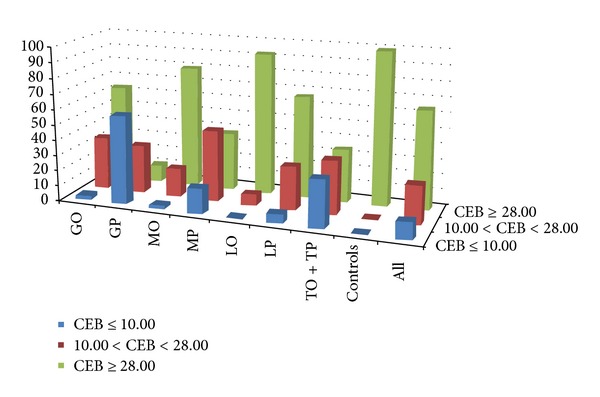
Distribution of relative frequency of CEB subgroups: percentage of individual cytological groups in strata of extent of anaerobic metabolism according to CEB (CEB ≥28.00 is in the reference range; CEB between 10.00 and 28.00 corresponds with advanced extent of anaerobic metabolism in serous CNS inflammations; CEB ≤10.00 corresponds with the oxidative burst of professional phagocytes).

Also the groups with MP and LP show a significant difference in comparison with controls (*P* < 0.001). But the same difference is between MP and GP, LP and GP (*P* < 0.001): this means that the values of CEB in GP are even significantly lower than in MP and LP (see Tables 2 and 3, Figures 2 and 4 again). MP can accompany cases of infection by intracellular bacteria, yeasts or molds, or malignant meningeal infiltration. Thus a decline of CEB to an anaerobic ratio can be expected in these cases, but they represent only a small proportion of the large group of MP. CEB values ≤10.00 are in 16.3% (237/1457) of MP and 5.8% (93/1610) of LP (see Figure 4). Furthermore, inflammatory process with the oxidative burst of macrophages is conditioned by the activation of specific immune components; therefore, it can fully develop after a certain interval. In case of severe immunodeficiency, it may not happen at all [13, 34, 35].

The interquartile range of CEB in the LP group is from 26.39 to 31.24, in the LO group from 30.14 to 31.97; these ranges correspond to serous inflammatory processes in CNS, which LP and LO usually accompany. Although the oxidative burst of phagocytes in terms of infection by intracellular bacteria in LP, LO can be sometimes expected, these cases are even less frequent than in MP (see Figure 4). Characteristic cytological picture of serous neuroinfections is LP, but we can also find GP, MP, and MO according to the stage. In all these cases the CEB is slightly decreased. Although the group of lymphocyte oligocytosis is not by any means identical with the control group, we found no difference of CEB values between LO and the control group. Lymphocyte oligocytosis is a cytological picture of autoimmune processes (such as multiple sclerosis) or chronic, nonpurulent, CNS inflammations (e.g., spirochaetal, neuroborreliosis, neurosyphilis). In these affections the oxidative burst of professional phagocytes is outstanding, which is supported by our findings.

The variance of CEB in TP in comparison with other groups still raises so far as this group is divided into two subgroups TO and TP, despite the small number of measurements (small number of measurements in the group with TO and TP is caused by low prevalence of these severe disorders in population). It can be seen in Table 4 and Figure 3.

In contrast to these results, the group with GO shows less significant difference of CEB in comparison with controls (*P* < 0.01) and no difference between GO and MO, LP. It may be caused by the fact that finding of GO is not usually caused by purulent neuroinfection. This cytological pattern appears in the initial stages of nonpurulent inflammations and in early stage of cerebral ischemia [36]. 

It should also be remembered that granulocyte pleocytosis and oligocytosis may rarely occur after subarachnoid hemorrhage, where the pseudopurulent noninfectious inflammation is induced by chemotactic and stimulatory effects of cytokines, the complement component C5a, and adhesion molecules; that is, both cytology and CEB correspond to the oxidative burst [29–33]. 

When we divide all values of CEB into three subgroups according to the following cut offs [5–7, 13–16]: 10.00 (cut off for the oxidative burst) and 28.00 (lower reference limit), we can construct Figure 4.

As can be seen in Table 2, Figures 2 and 4, the huge extent of CEB values is evident in the GP group: there are strongly negative values but also only slightly reduced values and values in reference ranges. CEB values ≤10.00 are in 57.8% (443/766) of GP, and CEB values within 10.00–28.00 are in 31.4% of GP. A part of CEB values above 10.00 probably corresponds to the early stages of serous neuroinfections, where nonspecific immune cells (neutrophils) may preponderate within a short time. Another part of these values may be caused by the so-called *preventive neuroprotection* when a systemic infection by neurotropic pathogens (e.g., typically the sepsis by *Neisseria meningitis*) is present. An activation of brain capillaries endothelium occurs, and then the permeability of blood-brain barrier increases—an influx of humoral immune components and immunocompetent cells to CSF raises to prepare this initially immune-privileged space for prospective invasion of pathogen and fully fledged inflammation process [3, 5, 7, 16, 36]. In this case, patterns imitating neuroinfection can be seen (GP or LP, hyperproteinorrhachia with an important share of immunoglobulins, acute phase proteins, complement, etc.), but there are no oxidative burst phagocytes yet. Just CEB enables distinguishing whether there is indeed an ongoing inflammation or whether it is absent (only the preventive neuroprotection) [5, 6, 14]. Another nonnegligible part of CEB values above 10.00 in GP is caused by an artificial blood admixture; 33.8% (259/766) of GP with CEB above 10.00 has erythrocyte count >100/1 *μ*L. 

Inclusion of data from patients with *secondary infection* after trauma or neurosurgical performance may bring a slight modification of results to our data set. In these cases the input of pathogens to the immune unprepared space causes slow acceleration of inflammation, and in addition there are often many other complications like production of glucocorticoids due to stress, a primary disease of CNS itself, a noninfectious inflammation after neurosurgical performance, a contamination of CNS by nonpathogenic microbes, usually a protective antibiotic therapy, polymorbidity, and so forth. These patients do not often develop an adequate inflammatory response, including cellularity of cerebrospinal fluid. A detection of metabolic manifestation of the oxidative burst by CEB significantly contributes to the solution of this difficult situation [5, 6, 37, 38]. 

Figure 4 shows that the highest percentage of CEB values less than 10.00 is in the GP group, the second largest in the group TO + TP; groups with LO, GO, and MO have very low share of these values.

To sum up, the “anaerobic shift” of CEB towards the values below the lower reference limit always reliably reveals the presence of pathology in CNS.

## 4. Conclusion

Advances in laboratory technologies affect an investigation of cerebrospinal fluid; processes occurring in CNS can be more accurately characterized by still new biomarkers measured at specialized centers. But we target the most fundamental energy parameters in CSF because they provide the first information about pathological processes in CNS to the physician, and they are generally available, low-cost, and irreplaceable. The Coefficient of Energy Balance enables more exact assessment of actual energy state in the CSF compartment than glucose and lactate alone, and not only does it specify and accelerate diagnostic and therapeutic decision, but it also determines the direction of further examinations. 

Using a large data set (*n* = 8183) we found statistically significant difference of CEB values in cytological groups accompanied by the oxidative burst of professional phagocytes. Our results confirm the ability of CEB to distinguish between variant pathologies in CNS, and thus support the benefit of CEB as an instrument for early differential diagnosis and early targeted therapy of CNS diseases. We believe that the combination of CEB and cytology in CSF examinations is crucial because cytology describes the presence of concrete cells, and CEB adds information about the functioning condition of these cells. Of course, it is also necessary to build diagnosis on all clinical and laboratory findings comprehensively, not only on one or several laboratory findings. 

CEB can reveal the presence of a serious disease of the central nervous system already at the level of the basic (urgent) analysis of cerebrospinal fluid. We are convinced that a calculation of CEB should be included into an urgent CSF analysis and automatically set in a Laboratory Information System. Further studies of CEB in relation to specific clinical diagnosis are needed to clarify sensitivity and specificity of CEB. Results from CSF open the possibility of using CEB for examinations of other body fluids.

## Figures and Tables

**Table 1:** Reference ranges of selected parameters of basic CSF examination (determined at our laboratory) [3, 4, 17–21].

Parameter	Age	Range	Unit
Energy metabolism			

*Q*_glu_		0.55–0.65	—
Lactate_CSF_		0.7–2.1	mmol/L

Cytology			

Number of leukocytes		0–4	/1 *µ*L
Cytological composition	Lymphocyte : monocyte = 7 : 3		

Permeability of blood-CSF barrier			

Total protein_CSF _(TP_CSF_)	0–2 weeks	450.0–1090.0	mg/L
	2–4 weeks	510.0–1010.0	
	1–3 months	240.0–650.0	
	3–6 months	230.0–370.0	
	6–12 months	170.0–350.0	
	1–10 years	160.0–310.0	
	11–18 years	160.0–400.0	
	19–40 years	240.0–490.0	
	41–50 years	270.0–600.0	
	51–60 years	290.0–670.0	
	61–70 years	260.0–790.0	

Albumin quotient (*Q*_alb_) *Q*_alb_ = [albumin_CSF_]/[albumin_blood_]	0–2 weeks	5.6–23.2	×10^*E*−3^
	2–4 weeks	7.6–16.4	
	1–3 months	2.3–10.6	
	3–6 months	2.0–4.8	
	6–12 months	1.4–4.5	
	1–10 years	1.0–4.5	
	11–18 years	1.0–5.0	
	19–30 years	1.7–5.7	
	31–40 years	1.8–6.2	
	41–50 years	2.0–7.2	
	51–60 years	2.1–8.9	
	61–70 years	2.2–9.9	

Inflammatory activity			

Beta-2-microglobulin_CSF_		0.2–2.0	mg/L
Intrathecal synthesis-IgG (Reiber)		0	%
Intrathecal synthesis-IgA (Reiber)		0	%
Intrathecal synthesis-IgM (Reiber)		0	%

**Table 2:** CEB with defined cytological syndromes.

Cytology	*N*	CEB						
		Median	Minimum	Maximum	1st quartile	3rd quartile	2.5th percentile	97.5th percentile
Controls	235	31.31	27.45	34.30	30.36	32.05	28.46	33.41
GO	64	29.55	7.00	35.03	26.24	31.12	10.06	33.26
GP	766	4.68	−2 996.50	33.55	−40.99	20.81	−1 158.70	31.75
LO	1 200	31.12	−1 249.00	35.11	30.14	31.97	24.06	33.35
LP	1 610	29.77	−3 980.50	37.36	26.39	31.24	−3.29	33.26
MO	2 699	30.45	−457.69	37.95	28.70	31.68	11.44	33.42
MP	1 457	25.96	−1 360.00	36.50	16.82	29.56	−52.05	32.66
TO + TP	152	21.80	−835.00	33.40	−1.66	29.52	−558.70	32.49

Descriptive statistics of CEB in 8 groups according to cytology; GO: granulocyte oligocytosis, GP: granulocyte pleocytosis, LO: lymphocyte oligocytosis, LP: lymphocyte pleocytosis, MO: monocyte oligocytosis, MP: monocyte pleocytosis, TO + TP: tumorous oligocytosis + pleocytosis.

**Table 3:** Results of post hoc multiple comparisons.

Compared groups	Level of significance
GO-GP	*P* < 0.01
GO-MO	No difference
GO-MP	*P* < 0.01
GO-LO	*P* < 0.01
GO-LP	No difference
GO-TO + TP	*P* < 0.01
GO-controls	*P* < 0.01
GP-MO	*P* < 0.001
GP-MP	*P* < 0.001
GP-LO	*P* < 0.001
GP-LP	*P* < 0.001
GP-TO + TP	*P* < 0.01
GP-controls	*P* < 0.001
MO-MP	*P* < 0.001
MO-LO	*P* < 0.01
MO-LP	*P* < 0.01
MO-TO + TP	*P* < 0.001
MO-controls	*P* < 0.01
MP-LO	*P* < 0.001
MP-LP	*P* < 0.001
MP-TO + TP	No difference
MP-controls	*P* < 0.001
LO-LP	*P* < 0.001
LO-TO + TP	*P* < 0.001
LO-controls	No difference
LP-TO + TP	*P* < 0.01
LP-controls	*P* < 0.001
TO + TP-controls	*P* < 0.001

The comparison of groups with each other; GO: granulocyte oligocytosis, GP: granulocyte pleocytosis, LO: lymphocyte oligocytosis, LP: lymphocyte pleocytosis, MO: monocyte oligocytosis, MP: monocyte pleocytosis, TO + TP: tumorous oligocytosis + pleocytosis.

**Table 4:** CEB with tumorous pleocytosis and tumorous oligocytosis.

Cytology	*N*	CEB						
		Median	Minimum	Maximum	1st quartile	3rd quartile	2.5th percentile	97.5th percentile
TO	31	31.13	−280.40	32.50	27.28	31.72	−223.50	32.49
TP	121	19.72	−835.00	33.40	−6.73	27.94	−671.73	32.49
TO + TP	152	21.80	−835.00	33.40	−1.66	29.52	−558.70	32.49
Controls	235	31.31	27.45	34.30	30.36	32.05	28.46	33.41

Descriptive statistics of CEB in the common TO + TP group versus separated TO and TP groups; TO: tumorous oligocytosis, TP: tumorous pleocytosis.
